# Delayed Diagnosis of a 17-Hydroxylase/17,20-Lyase Deficient Patient Presenting as a 46,XY Female: A Low Normal Potassium Level Can Be an Alerting Diagnostic Sign

**DOI:** 10.4274/jcrpe.3839

**Published:** 2017-06-01

**Authors:** Emine Çamtosun, Zeynep Şıklar, Serdar Ceylaner, Pınar Kocaay, Merih Berberoğlu

**Affiliations:** 1 Ankara University Faculty of Medicine, Department of Pediatric Endocrinology, Ankara, Turkey; 2 Intergen Genetics Center, Ankara, Turkey

**Keywords:** 17-hydroxylase deficiency, 46, XY disorder of sex development, diagnose, potassium

## Abstract

17-hydroxylase/17,20-lyase deficiency (17-OHD), a rare autosomal recessive defect in adrenal and gonadal steroidogenesis, causes absence of secondary sexual characteristics and frequently associated with hypertension and hypokalemia. Here, we report a 46,XY case who had normal potassium levels and no hypertension. Our patient was a 2.5-year-old female admitted with female external genitalia and inguinal swelling. Pathology of biopsy revealed that this gonad was a testis. Karyotype was 46,XY. She had no hypertension and no hypokalemia. Serum luteinizing hormone and follicle-stimulating hormone levels were high; testosterone, dehydroepiandrosterone sulfate, and androstenedione were low. Human chorionic gonadotrophin stimulation resulted in partial testosterone response. She was initially diagnosed as partial gonadal dysgenesis or testosterone synthesis defect. In her follow-up after noticing low normal potassium levels at age 9 years, progesterone level was measured and detected to be high. Adrenocorticotropic hormone-stimulated steroid measurements were consistent with 17-OHD. Genetic analyses revealed p. R96Q (c.287G>A) homozygous mutation on exon 1 of CYP17A1 gene. In conclusion, evaluation of 46,XY disorder of sex development patients must include serum potassium levels, and near low levels of potassium levels should also suggest 17-OHD despite absence of hypertension or remarkable hypokalemia. Testosterone synthesis defects must be excluded before establishing the diagnosis of partial gonadal dysgenesis.

## What is already known on this topic?

17-hydroxylase deficiency (17-OHD), a rare cause of congenital adrenal hyperplasia, is associated with hypertension and remarkable hypokalemia.

## What this study adds?

We emphasized that near low levels of potassium should suggest 17-OHD in a 46,XY disorder of sex development patient, thus may prevent diagnostic delay. We also compared phenotypes of literature cases that have the same mutation as our case.

## INTRODUCTION

17-hydroxylase/17,20-lyase deficiency (17-OHD) results from *CYP17A1* gene mutations. These mutations, disrupt steroidogenesis both in adrenals and gonads thus causing decreased production of glucocorticoids and sex steroids but increased mineralocorticoid precursors (1). CYP17A1 loss-of-function mutations can result in 17-OH or 17,20-lyase or combined enzyme deficiencies partially or completely (2). Over 100 mutations in the *CYP17A1* gene have been associated with combined 17-OH/17,20-lyase deficiency (OMIM 202110), including point mutations, small insertions or deletions, splice site alterations, and rarely large deletions (1). Isolated 17,20-lyase deficiency is rare and characterized with sex hormone deficiency without mineralocorticoid excess (3). P450 oxidoreductase (POR) and cytochrome b5 are also important for 17-OH/17,20-lyase enzyme activity. POR and cytochrome b5 deficiencies are among important differential diagnoses of 17-OHD (1).

The most common presentation of a 46,XX patient is an adolescent girl without secondary sexual characteristics or menses and showing a varying degree of low-renin hypertension and hypokalemia (1,4). Early diagnosis is easier in 46,XY patients who present with ambiguous genitalia or apparent female genitalia and an inguinal hernia/mass associated with hypertension and hypokalemia (1,4). Patients with an apparent female genitalia and who are not associated with the above features may go undiagnosed until adolescence or young adulthood and eventually present with lack of secondary sexual characteristics, hypertension and hypokalemia (2). The steroid profile of these patients shows low androgen, estrogen levels with high gonadotrophins and adrenocorticotropic hormone (ACTH). Cosyntropin stimulation testing results show low cortisol, dehydroepiandrosterone (DHEA), dehydroepiandrosterone sulfate (DHEA-S) and 17-OH progesterone but high, DOC and corticosterone levels and reveal the adrenal steroidogenic defect (1). Progesterone is also high in 17-OHD (5). Hypertensive patients have low renin and potassium levels, but 10%-15% of 17-OHD patients are normotensive at diagnosis (2).

Here we report a case with a p. R96Q (c.287G>A) mutation on exon 1 of *CYP17A1* gene who presented as a 46,XY disorder of sex development (DSD) with no hypertension and hypokalemia and who was misdiagnosed first as partial gonadal dysgenesis and subsequently diagnosed as 17-OHD via low normal levels of potassium and high levels of progesterone.

## CASE REPORT

This girl patient presented to our Pediatric Endocrinology Outpatient Unit at age 2.5 years for investigation of inguinal testis tissue on the right side. Her history revealed that she was born at term with a weight of 2670 grams and was registered as a girl. Her parents first noticed inguinal swelling while she was crying at age 5 months. The patient was admitted to a pediatric surgery unit in another hospital at age 2 years. When she was operated on for inguinal hernia, it was noticed that there was a gonad that seemed to be a testis. A gonadal biopsy was performed and pathologic evaluation revealed that the tissue was consistent with immature testis. The patient was referred to our hospital. She was the only child of non-consanguineous healthy parents. Her family history was unremarkable. On physical examination, her external genitalia had a female appearance and a vaginal opening was observed. Her height SDS was -0.81, blood pressure was normal (90/60 mmHg). Laboratory analyses revealed serum Na: 140 mEq/L (N: 135-145), K: 3.8 mEq/L (N: 3.5-5.5), follicle-stimulating hormone: 13.99 mIU/mL, luteinizing hormone: 2.19 mIU/mL, total testosterone (TT): 9.28 ng/dL, DHEA-S: 5.59 mcg/dL (N: <40), E2: 11.89 pg/mL, androstenedione: <0.03 ng/mL, dihydrotestosterone (DHT): 30.12 pg/mL. Human mammotropic gonadotropin (hMG)-stimulated estradiol level was 17.5 pg/mL, human chorionic gonadotropin (hCG)-stimulated testosterone was 50.04 ng/dL (partial response). Stimulated TT/DHT ratio was 2.05 (<12). Pelvic ultrasound revealed a 15x7.6x5 mm testis-like gonad in the right inguinal channel, and a 15x5x7 mm testis-like gonad in the left abdominal cavity. There were structures like prostate and seminal vesicles, but no Müllerian structures were seen. The patient’s karyotype was 46,XY and SRY was (+). Pathologic evaluation of the gonadal biopsy material in our hospital revealed that it was a testis tissue which included seminiferous tubules but no germ cell. The patient was diagnosed as having a partial gonadal dysgenesis or testosterone synthesis defect. At follow-up, psychiatric evaluation was compatible with female sexual identity and the local ethics committee decided that the patient be raised as a girl, with removal of the gonads because of potential malignancy. A gonadectomy was performed. On her follow-up, she had no hypertension. Her serum K levels were near low limits (between 3.7-3.9 mEq/L). At age 9 years, her serum Na was 142 mEq/L, K 3.7 mEq/L, plasma renin activity was 0.06 ng/mL/h (N: <17), progesterone level was high (9.57 ng/mL) (N: 0.07-0.52), and ACTH level was 40.78 pg/mL (N: 10-60). High-dose synacthen stimulation test (HDSST) revealed: basal cortisol: <0.4 mcg/dL (N: 8-21), DHEAS: 2.5 mcg/dL (N: 13-115), progesterone: 9 ng/mL (N: 0.07-0.52), 17-OH progesterone: 0.4 ng/dL (N: 0.03-0.9). Stimulated levels were 0.53 mcg/dL, 2.9 mcg/dL, 18.15 ng/mL, and 0.46 μg/dL, respectively. Low stimulated cortisol, DHEA-S, 17-0H progesterone, and high progesterone levels were consistent with 17-OH/17,20-lyase deficiency. Genetic analyses revealed p. R96Q (c.287G>A) homozygous mutation on exon 1 of *CYP17A1* gene which was previously reported as a cause of complete 17-hydroxylase/17,20-lyase deficiency. The patient was prescribed oral hydrocortisone (10 mg/m2/day) to prevent hypertension. On the last visit when she was 12.8 years old, her height SD was -1.75 and bone age was 8 years and 10 months. She had no hypertension. Her serum Na was 138 mEq/L and K was 4.5 mEq/L. Estrogen replacement therapy was planned on her follow-up.

## DISCUSSION

17-OHD is a rare form of congenital adrenal hyperplasia caused by mutations in the *CYP17A1* gene. 46,XX patients present with lack of secondary sexual characteristics or menses with hypertension and hypokalemia. 46,XY patients present with ambiguous genitalia or apparent female genitalia and inguinal hernia/mass associated with hypertension and hypokalemia. Müllerian structures (fallopian tubes, uterus, and upper third of vagina) are absent because Müllerian duct regression occurs due to normal production of Müllerian inhibitory factor from the testes. 17-OH and 17,20-lyase deficiencies can be diagnosed early and easily if hypertension and hypokalemia are associated with ambiguous genitalia or female external genitalia and an inguinal gonad in a 46,XY patient (1). However, hypertension and hypokalemia may not be seen in 10-15% of these patients and diagnosis may present difficulties in this group (2).

We reported here a case presenting as a 46,XY DSD patient with a low normal potassium level and no hypertension. Absence of hypertension and hypokalemia resulted in a delay in diagnosis until the patient reached the age of 9 years. At that time, noticing low normal levels of potassium levels brought to mind the correct diagnosis. Presence of a low stimulated cortisol, DHEA-S, 17-OH progesterone, and a high progesterone was consistent with a diagnosis of 17-OH/17,20-lyase deficiency. Genetic analyses revealed p. R96Q (c.287G>A) homozygous mutation on exon 1 of *CYP17A1* gene. p. R96Q mutation was reported before as associated with alterations in the steroid binding domain leading to complete 17α-OHD or combined 17-OH/17,20-lyase deficiency (2), but not reported from Turkey.

At the same amino acid site, first Laflamme et al (6) reported a homozygous novel missense mutation R96W caused by a C to T transition converting codon Arg96 (CGG) into a Trp (TGG) in exon 1 in two siblings with 46,XY DSD (14 and 9 years old). Both parents were heterozygous for this mutation. They showed that presence of R96W substitution almost completely abolished the activity of the mutant 17α-OH/17,20-lyase protein (6). These patients, similar to our patient, had no hypertension and hypokalemia.

Brooke et al (7) reported a novel missense homozygous R96Q mutation (same mutation of our case) in a 17-year-old 46,XX female patient who had presented with primary amenorrhea, sexual infantilism, and a malignant germ cell tumor. She had palmar and buccal hyperpigmentation, hypertension, and hypokalemia. Biochemical findings showed complete loss of 17-OH activity (7).

Athanasoulia et al (8) reported the same missense mutation in a 17-year-old 46,XY DSD patient who had presented with amenorrhea and no breast development and with mild diastolic hypertension. She was normokalemic. This patient also showed no breast development despite adequate estrogen replacement treatment for three years. The authors stated that the lack of breast development could be due to irreversible breast tissue alterations following high serum progesterone levels (8).

In a recent report, 4 affected XX siblings in an Arab family who had the same missense mutation were reported (9). The first sibling (17 years old) presented with abdominal pain and was diagnosed as a case of retroperitoneal malignant mixed germ cell tumor. She also had hypertension, primary amenorrhea, and lack of secondary sexual characteristics. One sibling (14 years old) presented with headache due to hypertension and pubertal delay. Two siblings (14 and 8 years old) were diagnosed with hypertension on a routine school check. All four patients had hypokalemia. They were treated with glucocorticoids and antihypertensive agents; three were also given estradiol for pubertal induction. Breast development in these patients was poor as also reported by Athanasoulia et al (8).

The phenotypic severity of combined 17α-OH/17,20-lyase deficiency varies depending on whether the activities of these enzymes are completely or partially lost according to the type and localization of the mutation. Alterations in the redox-partner binding site (e.g., p. R347H, p.R358Q, and p.E305G) lead to isolated 17,20-lyase deficiency (10), whereas mutations in the heme-binding site (e.g., p.R440C) or substrate binding pocket (e.g., p.S106P, D487_F489 deletion, duplication of I112, and p.R96Q) lead to complete 17α-OHD (2). Missense mutations in the steroid-binding domain, such as p.H373L, p. S106P, and p.R96Q, also result in combined 17α-OH /17,20-lyase deficiency (2).

The correlation between the CYP17A1 genotype and phenotype remains unclear. Patients who have the same mutations can have different presentations. Our case had no hypertension and no hypokalemia. The patient reported by Athanasoulia et al (8) had mild diastolic hypertension and normokalemia. On the other hand, the four siblings reported by Deeb and the case reported by Brooke et al (7) had notable hypertension and hypokalemia (9). Two patients with p. R96Q (c.287G>A) mutation were reported to have malignant germ cell tumor (7,9). This mutation may be associated with malignant germ cell tumor development in 46,XX patients. It is difficult to speculate that in CYP17A1 deficiency, the testes bear a malignant potential unless a dysgenesis exists. So, the decision for early gonadectomy should be cautious and better avoided. Gonadectomy should be postponed until pubertal ages so that the patient`s own consent and tumor surveillance can be suggested.

Some mutations were suggested as founder mutations for some populations: p.H373L (exon 6), p. Y329fs (exon 6), and D487_F489del (exon 8) mutations in Asian populations; pW406 and p.R362C (exon 6) mutations in Brazil; 4-bp duplication following Ile479 (exon 8) in Canadian Mennonites and Dutch Frieslanders; and p53(or54) del (exon1) in Japan (2).

*CYP17A1* gene mutations associated with 17-OHD previously reported from Turkey are: large deletions exons 1-6 (eight patients from two different families) (11,12), stop codon mutation p.Y27*(c.81C>A) in exon 1 (two patients from different families) (13,14), R239Q(G>A) exon not-known (one patient) (15), and a point mutation c.1307G>A (one patient) (16).

Patients who were diagnosed late showed poor breast development. It is suggested that high progesterone levels during pubertal development have irreversible effects on breast tissue. Spontaneous full breast development in patients who have normal progesterone levels and poor breast development in patients who have high progesterone levels support this suggestion (15). Accordingly, as a hypothesis, early diagnosis and treatment in these patients can prevent long-time exposure to high progesterone and breast development may be better in these cases. We will follow the breast development in our patient after estrogen replacement therapy. Deeb et al speculated that this particular mutation can have a specific adverse effect on breast development (9). Turan et al (15) also reported no improvement in breast development after estrogen replacement therapy in a 17-OHD patient who had another mutation (R239Q) in *CYP17A1* gene.

In conclusion, 17-OHD is a rare cause of 46,XY DSD. Although hypokalemic hypertension is a major component of 17-OHD, it is not seen in 10-15% of patients and due to this fact, diagnosis may be difficult and delayed. All 46,XY females should be first investigated for ACTH, PRA, HDSST, electrolyte status, and adrenal steroid profile before hCG test is performed. Low/near low levels of potassium and high progesterone levels should suggest 17-OHD despite absence of hypertension or remarkable hypokalemia. Early diagnosis and early treatment allow the induction of puberty at the appropriate time and can prevent hypertension and its complications.
